# In utero exposure to extra vitamin D from food fortification and the risk of subsequent development of gestational diabetes: the D-tect study

**DOI:** 10.1186/s12937-018-0403-5

**Published:** 2018-11-02

**Authors:** Amélie Keller, Maria Stougård, Peder Frederiksen, Fanney Thorsteinsdottir, Allan Vaag, Peter Damm, Ramune Jacobsen, Berit L. Heitmann

**Affiliations:** 1 Research Unit for Dietary Studies at The Parker Institute Bispebjerg and Frederiksberg Hospital, part of the Copenhagen University Hospital - The capital Region, Nordre Fasanvej 57, vej 8, entrance 11, 2000 Frederiksberg, Denmark; 2 Cardiovascular, Renal and Metabolic Disease (CVRM) Translational Medicine Unit, Early Clinical Development, IMED Biotech Unit, AstraZeneca, Gothenburg, Sweden; 3 Center for Pregnant Women with Diabetes, Department of Obstetrics, Rigshospitalet, Copenhagen, Denmark; 4 Institute of Clinical Medicine, Faculty of Health and Medical Sciences, University of Copenhagen, Copenhagen, Denmark; 5 Research Unit for Chronic Conditions, Center of Clinical Research and Prevention, Bispebjerg og Frederiksberg Hospital, Capital Region, Frederiksberg, Denmark; 6 The Boden Institute of Obesity, Nutrition, Exercise & Eating Disorders, University of Sydney, Sydney, Australia; 7 The National Institute of Public Health, University of Southern Denmark, Copenhagen, Denmark; 8 The Department of Public Health, Section for General Medicine, University of Copenhagen, Copenhagen, Denmark

**Keywords:** Vitamin D, Gestational diabetes mellitus, Public health epidemiology, Fetal programming, Food intake

## Abstract

**Background:**

The primary aim of this study was to assess whether exposure during fetal life to extra vitamin D from food fortification was associated with a reduction in the risk of subsequently developing gestational diabetes mellitus (GDM). Furthermore, we examined whether the effect of the vitamin D from fortification differed by women’s season of birth.

**Methods:**

This semi-ecological study is based on the cancellation in 1985 of the mandatory policy to fortify margarine with vitamin D in Denmark, with inclusion of entire national adjacent birth cohorts either exposed or unexposed to extra vitamin D in utero. The identification of GDM cases later in life among both exposure groups was based on the Danish national health registers. Logistic regression analyses generating odds ratios (ORs) and 95% confidence intervals (95% CIs) were performed.

**Results:**

Women who were prenatally exposed to the extra vitamin D from fortification tended to have a lower risk of subsequently developing GDM than unexposed women (OR 0.87, 95%CI 0.74,1.02, *P* = 0.08). When analyses were stratified by women’s season of birth, exposed women born in spring had a lower risk of developing GDM compared to unexposed subjects (OR 0.68, 95%CI 0.50,0.94, *p* = 0.02).

**Conclusion:**

This study suggests that prenatal exposure to extra vitamin D from mandatory fortification may lower the risk of developing gestational diabetes among spring-born women.

**Trial registration:**

This study is part of the D-tect project, which is registered on clinicaltrials.gov: NCT03330301.

**Electronic supplementary material:**

The online version of this article (10.1186/s12937-018-0403-5) contains supplementary material, which is available to authorized users.

## Background

Gestational diabetes mellitus (GDM) is defined as “carbohydrate intolerance of varying severity with onset or first recognition during pregnancy” [1]. In Europe, GDM is one of the most common pregnancy disorders with an estimated prevalence of 2–6% [2]. Women who develop GDM are more likely to experience pregnancy related complications such as pregnancy induced hypertension, obstructed labor and macrosomic newborns (birth weight > 4000 g) and have a high risk of later developing type 2 diabetes (T2D) [3]. Furthermore, offspring of GDM mothers are at increased risk of metabolic syndrome, GDM, T2D, obesity and cardiovascular disease later in life [2].

Obesity, increased maternal age, family history of T2D and previous delivery of a macrosomic infant are all known risk factors for GDM [4]. Nutritional risk factors such as high red and processed meat consumption as well as low-fiber and high glycemic index diets may increase the risk of GDM [5, 6]. In regards to the role of vitamin D on the risk of GDM development, discrepant evidence is present in the literature [7, 8], however recent evidence suggests that low blood vitamin D level during pregnancy could increase the risk of GDM, and vitamin D supplementation during pregnancy could improve GDM condition [9]. Vitamin D is a fat-soluble vitamin and a secosteroid obtained either from the diet (as D_2_ from vegetables and D_3_ from food such as oily fish and dairy products or fortified food and supplements); or through subcutaneous synthesis by exposure to sunlight [10]. The presence of nuclear vitamin D receptors and the vitamin D-activating 1-α-hydroxylase enzyme in the placenta suggests that vitamin D plays an important role during pregnancy [4] and may influence fetal development. Moreover, studies have also shown that low fetal or infancy vitamin D may influence later risk of disease development, such as type 1 diabetes (T1D) and pre-eclampsia, via so called early programming [11, 12]. However, no previous studies have examined if women with a lower fetal vitamin D exposure may be at higher subsequent risk of developing GDM than women with a higher fetal exposure to vitamin D.

In Denmark, fortification of margarine with vitamin A and D started in 1937 [13], and fortification with vitamin D was stopped by law on the 1st June 1985 [12, 14]. Between 1962 and 1985, margarine was fortified with 1.25 μg vitamin D/100 g of margarine, representing up to 29% of total dietary vitamin D intake (average 13%) [15]. To assess the association between exposure to extra vitamin D from food fortification in utero and the subsequent risk of developing GDM, all women born in Denmark two years prior and two years after the termination of the vitamin D fortification were included in this study. The primary aim of this study was to assess whether exposure during fetal life to extra vitamin D from food fortification was associated with a decreased risk of subsequently developing GDM. Furthermore, we examined whether the effect of the margarine fortification differed by women’s season of birth.

## Method

### Data sources

The Danish Civil Registration System (CRS) was used to retrieve information about the study population. Since April 1968, all people living in Denmark are registered by a 10-digit civil person registration (CPR) number in the CRS. Using this number, linkage of individual information from different registers and databases was possible. Information about antenatal care visits for all women with permanent residence in Denmark has been registered in the Danish Medical Birth Register (DMBR) since 1973 [16]. Since 1977, all non-psychiatric hospitals’ discharges, and since 1995, all emergency and outpatient departments’ discharges have been registered in the Danish National Patient Register (DNPR) [17]. Thus, using the CPR, information about pregnancy and offspring characteristics were retrieved from the DMBR, and the DNPR was used to identify diagnoses of GDM using the International Classification of Diseases (ICD) 10 code 024.4.

### Study population

**Fig. 1:**
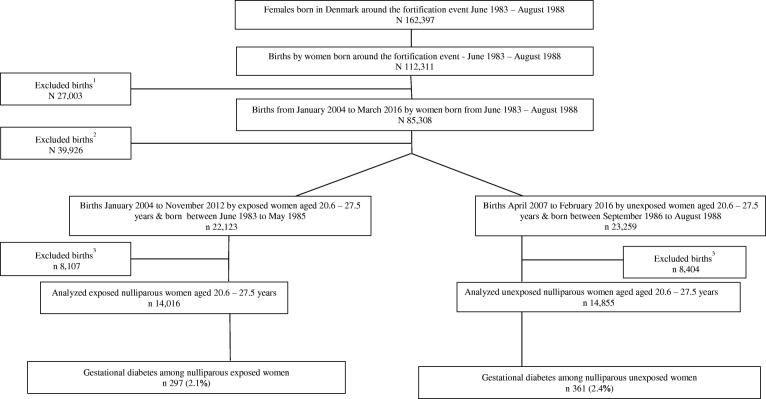
Flow chart of the study population, ^1^Exclusion criteria: Women born during the 15 months’ wash-out period from June 1985 to August 1986. ^2^Exclusion criteria: Women < 20.6 and > 27.5 years old or missing. ^3^Exclusion criteria: gestational weeks was < 22 weeks or missing, pre-pregnancy BMI was < 15 kg/m^2^ or > 50 kg/m2, women’s own birth weight was 0 g, multiple births, stillbirths, multiparous women

All women born in Denmark around the fortification termination, between June 1983 and August 1988 and who later gave birth were included in this study. Based on the termination of the margarine fortification policy on the 1st June 1985, women born between June 1983 and May 1985 were considered to have been prenatally exposed to extra vitamin D. To ensure the unexposed group was truly unexposed, a 15-month wash-out period was introduced, from June 1985 to September 1986, accounting for the 9 months of pregnancy and an additional 6 months to ensure vitamin D fortified margarine was no longer available on the Danish market or households. Thus, women born between September 1986 and August 1988 were considered to be unexposed (Fig.1).

The main analyses were restricted to nulliparous women who gave birth for the first time to a singleton infant between January 2004 and February 2016 after at least 22 weeks of gestation. In addition, women had to be between 20.6 and 27.5 years of age at time of delivery, to ensure same length of follow-up in both exposure groups. Therefore, women were excluded from the analyses if they had missing information on the following variables: age at delivery, whether it was a singleton or multiple births and offspring’s gestational age at delivery. Furthermore, women who were born during the 15-month’s wash-out period were excluded. Women with a pre-pregnancy BMI ≤15 or ≥ 50 kg/m^2^ were also excluded as erroneous data-entry in the DMBR was likely. Women whose own birth weight was registered as 0 g in the DMBR were also excluded as this likely reflected erroneous data entry in the DMBR.

### Outcome

In 2003, new national recommendations for screening and treatment of GDM, were implemented which improved GDM detection and management [18]. Since, selective screening for GDM has been recommended in Denmark to women with at least one of the following risk factors for GDM: previous GDM, previous birth to a large child (birth weight ≥ 4500 g), a pre-pregnancy body mass index (BMI) ≥27 kg/m^2^, a family history of diabetes, and glucosuria [18]. A 75 g 2-h oral glucose tolerance test (OGTT) is performed at 27–30 weeks and also in early pregnancy (14–20 weeks) for women with glucosuria, previous GDM or more than one of the aforementioned risk factors. The diagnostic criteria for GDM are a 2-h capillary blood or venous plasma glucose ≥9 mmol/L [18]. To ensure GDM diagnosis was harmonized all over Denmark, GDM diagnoses from 1st January 2004 and onwards were included in this study.

### Covariates

Information on smoking status during pregnancy, age at time of delivery, birth weight, pre-pregnancy BMI, singleton and multiple births, offspring gender, gestational age and parity were retrieved from the DMBR. Since 1997, data on smoking habits of pregnant women were collected in the DMBR. Smoking status during pregnancy was categorized into current smokers, former smokers (women who stopped smoking during or after the first trimester in the current pregnancy) and non-smokers. Pre-pregnancy BMI, based on self-reported height and weight at the first antenatal visit, was included in the DMBR since 2004. Pre-pregnancy BMI was categorized into the four following categories: underweight < 18.5 kg/m^2^, normal weight 18.5–24.9 kg/m^2^, overweight 25.0–29.9 kg/m^2^, obese ≥30 kg/m^2^. Women’s birth weight was categorized as follow: < 2500 g; 2500-4000 g; > 4000 g [19]. Offspring’s gestational age at delivery was categorized based on weeks + days as follow: < 37, 37 + 0 to 37 + 6, 38 + 0 to 38 + 6, 39 + 0 to 39 + 6, 40 + 0 to 40 + 6, 41 + 0 to 41 + 6 and ≥ 42 complete weeks of gestation [12]. Furthermore, size for gestational age (small for gestational age (SGA) (birth weight < 10th centile for gestational age and gender) and large for gestational age (LGA) (birth weight > 90th centile for gestational age and gender)) using Danish references was assessed [20]. Seasonality of birth in exposed/unexposed women was defined according to month of birth: Winter-born: November through January; Spring-born: February through April; Summer-born: May through July; Autumn-born: August through October. This categorization was based on the seasonal variation in serum 25(OH)D concentration among individuals from countries in northern latitudes [21].

### Statistical analyses

This study used entire birth cohorts of all women who were born in adjacent years around the termination of margarine fortification with vitamin D. As the women were unselected and, for their age, generally representative of the Danish population of women, in relation to both exposure status and development of GDM, we hypothesized that adjustment for covariates was not necessary. To confirm this hypothesis, a causal diagram, the directed acyclic graph (DAG), was used to identify possible confounders and to build the statistical regression analyses models [22]. Year of birth (as a proxy for exposure to vitamin D fortified margarine) was put into the model as the exposure of interest and GDM was the outcome variable of interest. The covariates mentioned above were put into the models as potential confounders. The presence or absence of a direct association between each variable-pair was assessed using a scientific framework built on theoretical and biological evidences from the literature [23]. This stepwise approach was used to draw a DAG on dagitty.net (Online-Only Additional file 1: DAG), from which the following statistical models were developed:

- Crude model: no adjustment
- Model 1: adjustment for women’s season of birth

Chi-squared test was used to test the differences between exposed and unexposed women for categorical variables and Mann-Whitney rank-sum test for skewed continuous data. Test for normality was not performed for age distribution, as it was a priori skewed because of the age restriction applied. The association between fetal exposure to vitamin D fortified margarine and the risk of developing GDM was examined by logistic regression generating odds ratios (ORs) and 95% confidence intervals (95% CIs). The effect of the vitamin D fortification on the fetus was expected to have most effect when stores are low, such as winter pregnancies; therefore, to evaluate whether women’s season of birth modified the association between prenatal vitamin D exposure and GDM, stratified analyses by women’s season of birth were performed.

### Sensitivity analyses

The prevalence of GDM has increased over the years and is higher at increased maternal age [24]. Therefore, trends in GDM incidence by maternal age, women’s year of birth and offspring’s year of birth were tested using Chi-squared test.

Prevalence of overweight and obesity among women of childbearing age, smoking habits during pregnancy and age at delivery have changed over time, and thus might have been different between exposed and unexposed women in this study. Therefore, sensitivity analyses adjusted for women’s pre-pregnancy BMI, smoking status in pregnancy and age at delivery were performed (Online-Only Additional file 1: Tables S1–S3).

The amount of bright sunshine hours during pregnancy has previously been reported to be an important variable to be adjusted for while analyzing exposure-outcome association in a similar setting [11], therefore, sensitivity analyses adjusted for total and trimestral gestational sunshine hours were performed. The data on bright sunshine hours were obtained from the Danish Meteorological Institute (DMI) [25]. Summary monthly sunshine hours during the nine months prior to the month of birth, bounded to each individual were calculated. First, second and third trimester sunshine hours were also calculated by summing recoded monthly sunshine hours during 1st, 2nd and 3rd thirds of these 9 months, respectively [11] (Online-Only Additional file 1: Tables S4a–S4 h).

Women born during the 15-month wash-out period were gradually less exposed to vitamin D from the fortified margarine. To test the additional hypothesis that these women have a higher risk of developing GDM than the exposed women, and a lower risk than the unexposed women, analyses with the three exposure groups were additionally performed (Online-Only Additional file 1: Tables S5a –S5b).

The statistical software package Stata 13 (StataCorp LP, College Station, Texas, 2013; https://www.stata.com/) was used for all data management and analyses. A level of significance as defined through *p*-values *p* < 0.05 was used.

## Results

There were 297 (2.1%) cases of GDM among women who were prenatally exposed to vitamin D fortified margarine (*N* = 14,016) compared to 361 (2.4%) GDM cases among the unexposed (N = 14,855) (*p* = 0.08) (Fig. 1). Maternal age at delivery was statistically slightly higher but the difference being clinically not relevant among the exposed compared to the unexposed with a median (5; 95 percentiles) of 25.2 (21.4; 27.3) and 25.1 (21.2; 27.3) years, respectively (*p* < 0.0001). Unexposed women were more likely to be former smokers and non-smokers compared to exposed women (5.6% vs. 5.2% and 81.3% vs.79.1%, respectively). Exposed women more often gave birth after 42 weeks of gestation (6.0% vs. 3.4%), and slightly more often had a pre-pregnancy BMI between 18.5 and 25 kg/m^2^ than unexposed women (60.7% vs. 59.6%) (*p* = 0.01). There was no difference in the distribution of the women’s season of birth between the two exposure groups (*p* = 0.8). Women born between 1983 and 1985 were exposed to more hours of bright sunshine during gestation than women born after the vitamin D fortification termination, median (5; 95 percentiles) 1108 (752;1311) and 1004 (772;1320) hours respectively (*p* < 0.0001) (Table 1).

**Table 1:** Characteristics of women prenatally exposed and unexposed to vitamin D fortified margarine and their offspring

	Exposed		Unexposed		*p*-value
	N	Median (5,95 percentiles)	N	Median (5,95 percentiles)	
Age at delivery^a,c^	14,016	25.2 (21.4;27.3)	14,855	25.1 (21.2;27.3)	< 0.0001
Gestational sunshine hours^a,c,e^	14,016	1108 (752;1311)	14,855	1004 (772;1320)	< 0.0001
Offspring’s birth weight (g) ^b,d^	14,016	3460 (2572;4270)	14,855	3455 (2560;4260)	0.3
		Percentage		Percentage	
Gestational diabetes mellitus^a^	297	2.1	361	2.4	0.08
Pre-pregnancy BMI^a,d^					0.01
< 18.5 kg/m^2^	636	4.5	800	5.4	
18.5–24.9 kg/m^2^	8514	60.7	8860	59.6	
25–29.9 kg/m^2^	2984	21.3	3154	21.2	
≥ 30 kg/m^2^	1882	13.4	2041	13.7	
Size for gestational age^a^					0.7
SGA	1741	12.4	1891	12.7	
NGA	11,299	80.7	11,961	80.6	
LGA	955	6.8	990	6.7	
Birth weight (g)^a,d^					0.8
< 2500	570	4.1	593	4.0	
2500–4000	11,536	82.3	12,274	82.6	
≥ 4000	1910	13.6	1988	13.4	
Smoking status during pregnancy^a,d^					< 0.0001
Current smoker	2170	15.8	1911	13.0	
Former smoker	715	5.2	827	5.6	
Non-smoker	10,890	79.1	11,935	81.3	
Season of birth^d^					0.8
Winter	3214	23.0	3465	23.3	
Spring	3568	25.5	3722	25.1	
Summer	3675	26.2	3875	26.1	
Autumn	3559	25.4	3793	25.5	
Offspring’s gestational age at delivery (weeks)^b,d^					< 0.0001
< 37	871	6.2	892	6.0	
37 + 0–37 + 6	699	5.0	727	4.9	
38 + 0–38 + 6	1688	12.0	1751	11.8	
39 + 0–39 + 6	3068	21.9	3150	21.2	
40 + 0–40 + 6	4033	28.8	4423	29.8	
41 + 0–41 + 6	2819	20.1	3414	23.0	
≥ 42	838	6.0	498	3.4	
Offspring’s gender^b,d^					0.02
Female	6920	49.4	7128	48.0	
Male	7096	50.6	7727	52.0	

^a^Characteristics of women prenatally exposed and unexposed to vitamin D fortified margarine and ^b^ their offspring

^c^tested by Mann-Whitney rank-sum test and ^d^ tested by X^2^-test

^e^Summary monthly sunshine hours during the nine months prior to the month of birth, bounded to each individual

Women prenatally exposed to the vitamin D fortification tended to have a lower risk of developing GDM than unexposed women (Crude model: OR 0.87, 95%CI 0.74,1.02, *p* = 0.08; Model 1: OR 0.87, 95%CI 0.74,1.02, *P* = 0.08) (Table 2). Further adjustment for women born SGA or LGA to account for differences in birth weight adjusted for gestational age gave essentially similar results (data not shown). When analyses were stratified by women’s season of birth, exposed women born in spring (winter pregnancies) had a lower risk of developing GDM compared to those unexposed to extra vitamin D (OR 0.68, 95%CI 0.50,0.94, *p* = 0.02) (Table 3).

**Table 2:** Crude and adjusted odds ratio (OR) and 95% confidence intervals (95%CI) for gestational diabetes mellitus among women prenatally exposed and unexposed to vitamin D fortified margarine

Exposure		Crude^a^			Model 1^a^		
	N	OR	95%CI	*p*-value	OR	95%CI	*p*-value
Unexposed	14,855	1			1		
Exposed	14,016	0.87	0.74, 1.02	0.08	0.87	0.74, 1.02	0.08

^a^ *Crude model: no adjustment; Model 1a: adjustment for women’s season of birth*

**Table 3:** Crude odds ratio (OR) and 95% confidence intervals (95%CI) for gestational diabetes mellitus among women prenatally exposed and unexposed to vitamin D fortified margarine stratified by women’s season of birth

Seasons of birth^a^		N	% GDM	Crude		
	Exposure			OR	95%CI	*p*-value
Winter	Unexposed	3465	2.3	1		
	Exposed	3214	2.3	0.98	0.71, 1.35	0.92
Spring	Unexposed	3722	2.6	1		
	Exposed	3568	1.8	0.68	0.50, 0.94	0.02
Summer	Unexposed	3875	2.5	1		
	Exposed	3675	2.0	0.83	0.61, 1.13	0.23
Autumn	Unexposed	3793	2.4	1		
	Exposed	3559	2.4	1.02	0.75, 1.38	0.91

^a^Winter: November to January; Spring: February to April; Summer: May to July; Autumn: August to October

Results from sensitivity analyses were essentially similar to the main results (Online-Only Additional file 1: Tables S1-S4). Women born during the wash-out period tended to have a higher risk of developing GDM compared to exposed women (Crude model: OR 1.18, 95%CI 0.99,1.40, *p* = 0.07; Model 1: OR 1.17, 95%CI 0.98,1.40, p = 0.08) and a similar risk compared to the unexposed women (Crude model: OR 1.02, 95%CI 0.87,1.21, *p* = 0.78; Model 1: OR 1.02, 95%CI 0.86,1.21, *p* = 0.83), however the differences were not significant (Online-Only Additional file 1: Tables S5a –S5b).

There was a higher GDM incidence with higher age at delivery (*p* = 0.004), and both women’s (*p* = 0.04) and offspring’s years of birth (*p* < 0.0001).

## Discussion

This study suggests that prenatal exposure to extra vitamin D from mandatory fortification may lower the risk of developing gestational diabetes among spring-born women. This finding suggests that the extra vitamin D from fortification may have been of particular benefit to offspring of mothers who were pregnant during most of the dark months when vitamin D skin synthesis is low or null, and when diet or supplementation are the sole sources of vitamin D.

Few previous studies have examined association between early exposure to vitamin D and the development of diabetes later in life. One previous longitudinal study found an association between low concentrations of 25(OH)D in pregnant mothers and fasting insulin concentrations and insulin resistance in their children at 9.5 years of age [26]. As glucose homeostasis variables tend to track from childhood to adulthood [27], 25(OH)D concentration during fetal life might also influence glucose homeostasis variables in childhood and may predict the development of diabetes in adulthood. In regards to T1D, a meta-analysis has concluded that vitamin D supplementation in early childhood may be associated with a lower risk of developing of T1D [28]; however, the risk of developing T1D was not different for subjects exposed and unexposed to extra vitamin D from fortified margarine in our previous study [29]. The discrepant findings between exposure to vitamin D fortification during fetal life on the risk of developing T1D and GDM might be due to a possible different genetic-environmental risk factors ratio between the two diseases; GDM being more susceptible to lifestyle related risk factors than T1D.

Several potential mechanisms could explain our finding of a potential protective effect of exposure to vitamin D in early life on the subsequent development of GDM. Multiple factors affecting early growth may induce changes in the structure and function of certain organs and tissues such as the pancreas [30]. Results from animal models suggest that maternal vitamin D restriction may lead to insulin dysregulation inducing a compensatory increase in pancreatic beta-cell mass in the offspring [30]. Another mechanism might be related to the mediating role of vitamin D on the association between amino acids supply and fetal growth [31]. Animal models suggest that maternal protein restriction may cause chronic disease in adulthood among first and second offspring generations [30]. Already in 1992, Hales & Barker stated that β-cell growth and development as well as insulin secretion until late fetal life are controlled by amino acids supply [32]. Therefore, an unanswered question is whether vitamin D may be involved in the amino acids supply to the fetus. A recent human study, suggested that placental amino acid transport may be regulated by maternal vitamin D and vitamin D-binding protein [33]. Hence, exposure to extra vitamin D during fetal life may promote adequate β-cell growth by mediating placental amino acid transport and decrease the risk of developing diabetes in later life [33]. Moreover, vitamin D supply might be especially important during the second gestational trimesters as animal studies have confirmed that it is an important period for development and biochemical maturation of β-cells, however these findings were not confirmed in a human study [34].

There is increasing evidence for a potential role of inflammation in the pathogenesis of T2D and GDM [35]. During pregnancy, vitamin D receptors and 1,25(OH)_2_D_3_ have been described to play a role in the adaptive and innate immune system as well as in the secretion of insulin by the pancreatic β-cells [36]. Therefore, exposure to extra gestational vitamin D might reduce the risk of GDM by modulating the innate immune system, particularly when vitamin D skin synthesis is low or absent. From 1937 to 1985, margarine was fortified with both vitamins D and A; and from 1985 only with vitamin A [12]. Therefore, the potential protective effect of in utero exposure to vitamin D fortified margarine on the development of GDM seen in this study may be due to additive, synergistic or antagonist interactions between vitamins A and D. It has been reported that both vitamins may be involved in the development and functioning of human fetal pancreas. However, more studies are needed to explain these mechanisms and their potential role in the development of diabetes in humans [14].

### Methodological considerations

This study is the first to examine if fetal exposure to extra vitamin D may influence GDM development. The inclusion of all women from entire birth cohorts of the whole Danish population was made possible by the complete registration of every citizen via a CPR number into the Danish national health registers. By separating women from exposure groups by a specific point in time, selection bias was minimized. However, residual confounding cannot be excluded and in the following sections several potential confounders are discussed.

#### Age at delivery

Older women have a higher risk of developing GDM [24] and in this study we also saw a linear association between higher risk of GDM and older age at delivery. Age at delivery in our study was slightly higher in exposed compared to unexposed women, which advocated for a true association between the prenatal exposure to vitamin D fortification and GDM risk. However, women included in this study were relatively young and are not representative of all women that develop GDM.

#### Trends in birth weight

Increased birth weight has been associated with later onset of GDM [37]. However, no secular trend in birth weight between 1983 and 1988 were seen in this study (Online-Only Additional file 1: Table S6).

#### Overweight and obesity

The prevalence of overweight and obesity, which are strong risk factors of GDM [4], is increasing among women of childbearing age, particularly among young women [38]. Our sensitivity analyses that adjusted for pre-pregnancy BMI gave similar results as analyses without adjustment, suggesting that maternal BMI was not associated with the development of GDM in the present study. However, residual confounding related to fat mass or fat distribution, which we did not have information on, is still a possibility.

#### Diet and vitamin D supplementation

No significant changes in margarine consumption in Denmark in the period analyzed have been reported [39]. Furthermore, there were no changes in recommendations regarding vitamin D intake or vitamin supplementation during pregnancy between 1983 and 1988 [12]. Therefore, it is unlikely that these factors influenced our results.

#### Bright sunshine hours as a source of vitamin D

Sensitivity analyses including adjustment for individual bright sunshine hours showed similar results as the main analyses without adjustment, suggesting that the effect of the vitamin D fortification during gestation on later risk of GDM observed in this study was not confounded by vitamin D synthesized in the skin.

#### Smoking

We did not have data on smoking by the mothers of the women included, however, it is estimated that about 40% of all women smoked during pregnancy between 1983 and 1993 [40], and that the proportion of smokers, thus, did not differ between the exposed and unexposed group. On the other hand, the prevalence of women smoking during pregnancy has since been decreasing [41], and as smoking has been shown to be associated with GDM risk [42], such change in smoking habits might have influenced our results. However, sensitivity analyses adjusted for pregnancy smoking habits showed similar results as analyses without adjustment, suggesting that smoking during pregnancy did not confound the association observed.

#### Assisted reproductive technology (ART)

In Europe, the use of ART has been increasing over the years and an increased risk of GDM following ART treatment has been reported [43]. The chance that women conceived with the help of ART in our study was most likely slightly larger for unexposed than exposed women, and hence the use of ART could potentially have confounded the association observed. However, most women undergoing ART treatment in Denmark are over 30 years old and in the present study the oldest women were 27.5 years at time of delivery. Therefore, the risk of potential confounding by ART is expected to be minimal.

#### Induced labor

A more aggressive policy towards inducing delivery after 41+ weeks was introduced in Denmark around 2009 [44], potentially leading the unexposed group to have less “time in pregnancy” to develop/or have a diagnosis of GDM. However, GDM is rarely diagnosed after term since routine screening is generally performed at the latest at 27–30 weeks of gestation [18]. When analyses were restricted to women delivering before 41 weeks of gestation, similar results were found (data not shown).

#### GDM prevalence

In Denmark, the prevalence of GDM is relatively low on a global scale and has increased from 1.7 to 2.9% between 2004 and 2012 [24]. Therefore, although the difference between the two exposure groups (2.1% vs. 2.4%) may seem small, it might still be clinically relevant.

In this study, GDM risk increased with women’s year of birth: from 2.0% in those born in 1983 to 2.9% in those born in 1988. This suggests that secular trends in GDM risk in Denmark may be present, making it difficult to disentangle the specific effects related to vitamin D fortification.

#### GDM at subsequent pregnancies

Women who develop GDM during their first pregnancy are at increased risk of developing GDM in their subsequent pregnancies [3]. Therefore, analyses to examine the risk of GDM in the second pregnancy, conditional on whether GDM was present in the first pregnancy, among women with two pregnancies, was planned. However, no women met the criteria.

#### Diagnosis of GDM

The diagnostic criteria for GDM did not change during the study period and by using the DMBR and DNPR, selection bias and risk of loss to follow-up could be avoided. Universal health coverage in Denmark secures that all Danish residents have access to healthcare regardless of their ethnic background or socioeconomic status. All deliveries, either at hospitals or at home with a midwife attending from the regional hospitals, are recorded in the registers. Therefore, most GDM diagnoses are registered in the DNPR.

Finally, we cannot exclude that other environmental and/or societal changes, such as an increase in the exposure to endocrine disrupting chemicals [45], may have influenced our results for instance through epigenetic changes influencing lifelong health and disease by modifying inflammatory molecular pathways and the immune response [46].

## Conclusion

This study suggests that prenatal exposure to extra vitamin D from mandatory fortification may lower the risk of developing gestational diabetes among spring-born women, eg. from winter pregnancies, when the small extra amount of vitamin D from fortification seemed particularly beneficial.

Our results may have public health relevance as they demonstrate that mothers consuming extra vitamin D from food fortification had daughters who were at lower subsequent risk of developing GDM. However, recommendations for vitamin D supplementation or fortification cannot be provided on the basis of this study and would require long-term randomized controlled trials.

## Additional file

Additional file 1: In utero exposure to extra vitamin D from food fortification and the risk of subsequent development of gestational diabetes: the D-tect study. (DOCX 1837 kb)
