# Supplementary material for: In utero exposure to extra vitamin D from food fortification and the risk of subsequent development of gestational diabetes: the D-tect study
Source: Nutr J. 2018 Nov 2;17:100. doi: 10.1186/s12937-018-0403-5 (PMC6215342; doi:10.1186/s12937-018-0403-5)
Supplement: Supplementary file 1 — In utero exposure to extra vitamin D from food fortification and the risk of subsequent development of gestational diabetes: the D-tect study. (DOCX 1837 kb) [file 12937_2018_403_MOESM1_ESM.docx]

**Online-Only additional file 1: DAG**

***Unadjusted model***


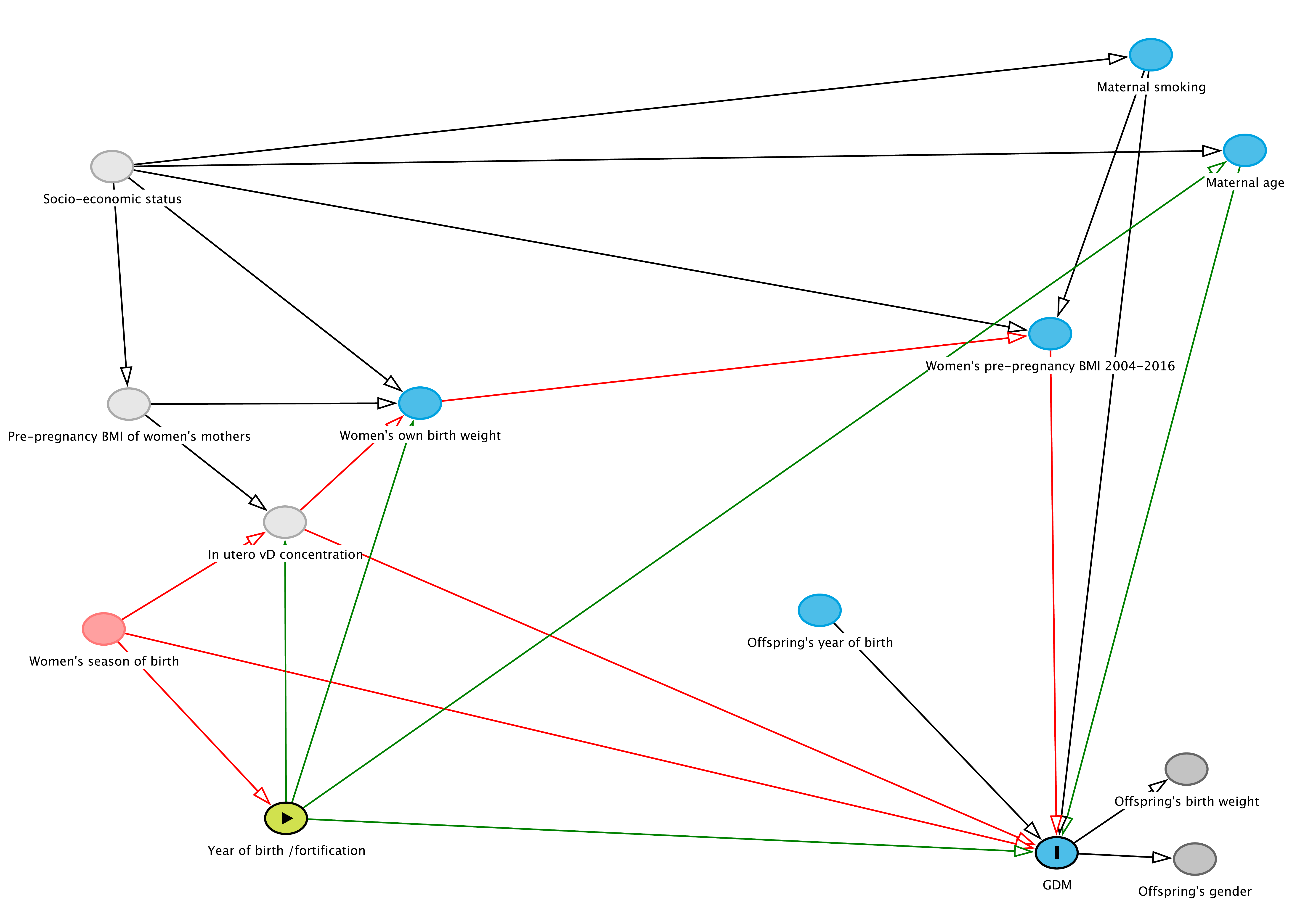


Made on http://dagitty.net/

***Adjusted model***


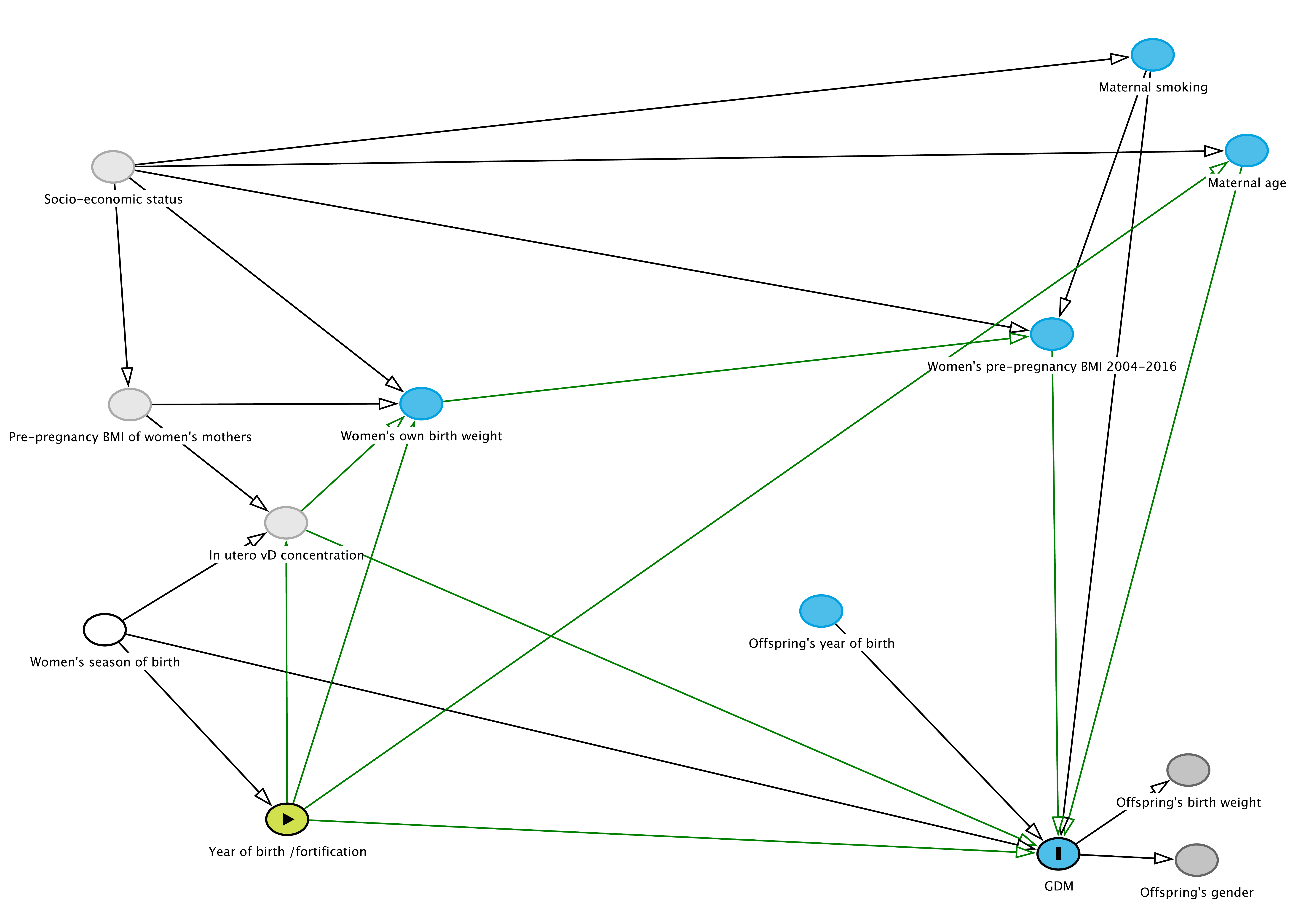


Made on http://dagitty.net/

**Legend**

exposure


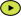


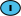
outcome


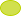
ancestor of exposure


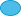
ancestor of outcome


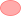
ancestor of exposure and outcome


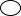
adjusted variable


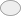
unobserved (latent)


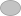
other variable


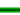
causal path


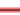
biasing path

#### Table S1a: Odds ratio (OR) and 95% confidence intervals (95%CI) for gestational diabetes mellitus among women prenatally exposed and unexposed to vitamin D fortified margarine adjusted for women’s pre-pregnancy BMI

|  |  | **Crude^*^** | | | **Model 1^*^** | | | **Model 2^*^** | | |
| --- | --- | --- | --- | --- | --- | --- | --- | --- | --- | --- |
| **Exposure** | **N** | **OR** | **95%CI** | **p-value** | **OR** | **95%CI** | **p-value** | **OR** | **95%CI** | **p-value** |
|  |  |  |  |  |  |  |  |  |  |  |
| Unexposed | 14,855 | 1 |  |  | 1 |  |  | 1 |  |  |
| Exposed | 14,016 | 0.87 | 0.74, 1.02 | 0.08 | 0.87 | 0.75, 1.02 | 0.09 | 0.87 | 0.75, 1.02 | 0.09 |

#### ^*^ Crude model: no adjustment; model 1: adjustment for pre-pregnancy BMI; Model 2: Model 1 + adjustment for women’s season of birth

**Table S1b: Crude odds ratio (OR) and 95% confidence intervals (95%CI) for gestational diabetes mellitus among women prenatally exposed and unexposed to vitamin D fortified margarine stratified by women’s pre-pregnancy BMI**

|  |  | **BMI <25** | | |  | **BMI ≥ 25** | | |
| --- | --- | --- | --- | --- | --- | --- | --- | --- |
| **Exposure** | **N** | **OR** | **95%CI** | **p-value** | **N** | **OR** | **95%CI** | **p-value** |
|  |  |  |  |  |  |  |  |  |
| Unexposed | 9,660 | 1 |  |  | 5,195 | 1 |  |  |
| Exposed | 9,150 | 0.87 | 0.67, 1.14 | 0.31 | 4,866 | 0.87 | 0.72, 1.06 | 0.16 |

#### Table S2a: Odds ratio (OR) and 95% confidence intervals (95%CI) for gestational diabetes mellitus among women prenatally exposed and unexposed to vitamin D fortified margarine adjusted for women’s age at delivery

|  |  | **Crude^*^** | | | **Model 1^*^** | | | **Model 2^*^** | | |
| --- | --- | --- | --- | --- | --- | --- | --- | --- | --- | --- |
| **Exposure** | **N** | **OR** | **95%CI** | **p-value** | **OR** | **95%CI** | **p-value** | **OR** | **95%CI** | **p-value** |
|  |  |  |  |  |  |  |  |  |  |  |
| Unexposed | 14,855 | 1 |  |  | 1 |  |  | 1 |  |  |
| Exposed | 14,016 | 0.87 | 0.74, 1.02 | 0.08 | 0.86 | 0.73, 1.00 | 0.05 | 0.86 | 0.73, 1.00 | 0.06 |

#### ^*^ Crude model: no adjustment; model 1: adjustment for maternal age at delivery; Model 2: Model 1 + adjustment for women’s season of birth

**Table S2b: Crude odds ratio (OR) and 95% confidence intervals (95%CI) for gestational diabetes mellitus among women prenatally exposed and unexposed to vitamin D fortified margarine stratified by women’s age at delivery**

|  |  | **Age 20** | | |  | **Age 21** | | |  | **Age 22** | | | |
| --- | --- | --- | --- | --- | --- | --- | --- | --- | --- | --- | --- | --- | --- |
| **Exposure** | **N** | **OR** | **95%CI** | **p-value** | **N** | **OR** | **95%CI** | **p-value** | **N** | **OR** | **95%CI** | | **p-value** |
|  |  |  |  |  |  |  |  |  |  |  |  | |  |
| Unexposed | 487 | 1 |  |  | 1,198 | 1 |  |  | 1,496 | 1 |  | |  |
| Exposed | 358 | 0.91 | 0.25, 3.23 | 0.88 | 1,024 | 0.52 | 0.27, 1.00 | 0.05 | 1,281 | 0.49 | 0.26, 0.92 | | 0.03 |
|  |  | **Age 23** | | |  | **Age 24** | | | **N** | **Age 25** | | | |
| **Exposure** | **N** | **OR** | **95%CI** | **p-value** | **N** | **OR** | **95%CI** | **p-value** |  | **OR** | **95%CI** | **p-value** | |
|  |  |  |  |  |  |  |  |  |  |  |  |  | |
| Unexposed | 1,828 | 1 |  |  | 2,250 | 1 |  |  | 2,692 | 1 |  |  | |
| Exposed | 1,660 | 0.67 | 0.41, 1.09 | 0.11 | 2,134 | 1.33 | 0.87, 2.02 | 0.19 | 2,735 | 0.74 | 0.53;1.02 | 0.07 | |
|  |  | **Age 26** | | |  | **Age 27** | | |  |  |  |  |  |
| **Exposure** | **N** | **OR** | **95%CI** | **p-value** | **N** | **OR** | **95%CI** | **p-value** |  |  |  |  |  |
|  |  |  |  |  |  |  |  |  |  |  |  |  |  |
| Unexposed | 3,113 | 1 |  |  | 1,791 | 1 |  |  |  |  |  |  |  |
| Exposed | 3,100 | 0.99 | 0.72, 1.37 | 0.96 | 1,724 | 1.09 | 0.72, 1.64 | 0.69 |  |  |  |  |  |

#### Table S3a: Odds ratio (OR) and 95% confidence intervals (95%CI) for gestational diabetes mellitus among women prenatally exposed and unexposed to vitamin D fortified margarine adjusted for women’s smoking status during pregnancy

|  |  | **Crude^*^** | | | **Model 1^*^** | | | **Model 2^*^** | | |
| --- | --- | --- | --- | --- | --- | --- | --- | --- | --- | --- |
| **Exposure** | **N** | **OR** | **95%CI** | **p-value** | **OR** | **95%CI** | **p-value** | **OR** | **95%CI** | **p-value** |
|  |  |  |  |  |  |  |  |  |  |  |
| Unexposed | 14,855 | 1 |  |  | 1 |  |  | 1 |  |  |
| Exposed | 14,016 | 0.87 | 0.74, 1.02 | 0.08 | 0.87 | 0.74, 1.02 | 0.08 | 0.87 | 0.74, 1.02 | 0.08 |

#### ^*^ Crude model: no adjustment; model 1: adjustment for maternal smoking; Model 2: Model 1 + adjustment for women’s season of birth

**Table S3b: Crude odds ratio (OR) and 95% confidence intervals (95%CI) for gestational diabetes mellitus among women prenatally exposed and unexposed to vitamin D fortified margarine stratified by women’s smoking status during pregnancy**

|  |  | **Smoker** | | |  | **Previous smoker** | | |  | **Non-smoker** | | |
| --- | --- | --- | --- | --- | --- | --- | --- | --- | --- | --- | --- | --- |
| **Exposure** | **N** | **OR** | **95%CI** | **p-value** | **N** | **OR** | **95%CI** | **p-value** | **N** | **OR** | **95%CI** | **p-value** |
|  |  |  |  |  |  |  |  |  |  |  |  |  |
| Unexposed | 1,911 | 1 |  |  | 827 | 1 |  |  | 11,935 | 1 |  |  |
| Exposed | 2,170 | 0.77 | 0.51,1.16 | 0.21 | 715 | 0.91 | 0.50,1.68 | 0.77 | 10,890 | 0.88 | 0.74,1.05 | 0.17 |

#### Table S4a: Odds ratio (OR) and 95% confidence intervals (95%CI) for gestational diabetes mellitus (GDM) among women prenatally exposed and unexposed to vitamin D fortified margarine adjusted for amount of bright sunshine hours during pregnancy

|  |  | **Crude^*^** | | | **Model 1^*^** | | |
| --- | --- | --- | --- | --- | --- | --- | --- |
| **Exposure** | **N** | **OR** | **95%CI** | **p-value** | **OR** | **95%CI** | **p-value** |
|  |  |  |  |  |  |  |  |
| Unexposed | 14,855 | 1 |  |  | 1 |  |  |
| Exposed | 14,016 | 0.87 | 0.74, 1.02 | 0.08 | 0.88 | 0.75, 1.03 | 0.11 |

#### ^*^ Crude model: no adjustment; model 1: adjustment for amount of bright sunshine hours during whole pregnancy

#### Table S4b: Odds ratio (OR) and 95% confidence intervals (95%CI) for gestational diabetes mellitus (GDM) among women prenatally exposed and unexposed to vitamin D fortified margarine adjusted for amount of bright sunshine hours during pregnancy stratified by women’s season of birth

|  |  | **N** | **Model 1^*^** | | |
| --- | --- | --- | --- | --- | --- |
| **Seasons of birth**^†^ | **Exposure** |  | **OR** | **95%CI** | **p-value** |
| Winter |  |  |  |  |  |
|  | Unexposed | 3465 | 1 |  |  |
|  | Exposed | 3214 | 1.02 | 0.73, 1.43 | 0.89 |
| Spring |  |  |  |  |  |
|  | Unexposed | 3722 | 1 |  |  |
|  | Exposed | 3568 | 0.74 | 0.54, 1.03 | 0.08 |
| Summer |  |  |  |  |  |
|  | Unexposed | 3875 | 1 |  |  |
|  | Exposed | 3675 | 0.82 | 0.60, 1.12 | 0.21 |
| Autumn |  |  |  |  |  |
|  | Unexposed | 3793 | 1 |  |  |
|  | Exposed | 3559 | 1.12 | 0.81, 1.53 | 0.52 |

#### ^*^ Model 1: adjustment for amount of bright sunshine hours during whole pregnancy

^†^Winter: November to January; Spring: February to April; Summer: May to July; Autumn: August to October

####

#### Table S4c: Odds ratio (OR) and 95% confidence intervals (95%CI) for gestational diabetes mellitus (GDM) among women prenatally exposed and unexposed to vitamin D fortified margarine adjusted for amount of bright sunshine hours during the first trimester of pregnancy

|  |  | **Crude ^†^** | | | **Model 1^†^** | | |
| --- | --- | --- | --- | --- | --- | --- | --- |
| **Exposure** | **N** | **OR** | **95%CI** | **p-value** | **OR** | **95%CI** | **p-value** |
|  |  |  |  |  |  |  |  |
| Unexposed | 14,855 | 1 |  |  | 1 |  |  |
| Exposed | 14,016 | 0.87 | 0.74, 1.02 | 0.08 | 0.87 | 0.75, 1.02 | 0.09 |

#### ^†^ Crude model: no adjustment; model 1: adjustment for amount of bright sunshine hours during 1^st^ trimester of pregnancy

#### Table S4d: Odds ratio (OR) and 95% confidence intervals (95%CI) for gestational diabetes mellitus (GDM) among women prenatally exposed and unexposed to vitamin D fortified margarine adjusted for amount of bright sunshine hours during the first trimester of pregnancy stratified by women’s season of birth

|  |  | **N** | **Model 1^†^** | | |
| --- | --- | --- | --- | --- | --- |
| **Seasons of birth^¥^** | **Exposure** |  | **OR** | **95%CI** | **p-value** |
| Winter |  |  |  |  |  |
|  | Unexposed | 3465 | 1 |  |  |
|  | Exposed | 3214 | 0.98 | 0.71, 1.36 | 0.93 |
| Spring |  |  |  |  |  |
|  | Unexposed | 3722 | 1 |  |  |
|  | Exposed | 3568 | 0.81 | 0.57, 1.13 | 0.22 |
| Summer |  |  |  |  |  |
|  | Unexposed | 3875 | 1 |  |  |
|  | Exposed | 3675 | 0.83 | 0.61, 1.13 | 0.23 |
| Autumn |  |  |  |  |  |
|  | Unexposed | 3793 | 1 |  |  |
|  | Exposed | 3559 | 1.02 | 0.75, 1.38 | 0.91 |

#### ^†^ Model 1: adjustment for amount of bright sunshine hours during whole pregnancy

^¥^Winter: November to January; Spring: February to April; Summer: May to July; Autumn: August to October

#### Table S4e: Odds ratio (OR) and 95% confidence intervals (95%CI) for gestational diabetes mellitus (GDM) among women prenatally exposed and unexposed to vitamin D fortified margarine adjusted for amount of bright sunshine hours during the second trimester of pregnancy

|  |  | **Crude ^†^** | | | **Model 1^†^** | | |
| --- | --- | --- | --- | --- | --- | --- | --- |
| **Exposure** | **N** | **OR** | **95%CI** | **p-value** | **OR** | **95%CI** | **p-value** |
|  |  |  |  |  |  |  |  |
| Unexposed | 14,855 | 1 |  |  | 1 |  |  |
| Exposed | 14,016 | 0.87 | 0.74, 1.02 | 0.08 | 0.87 | 0.74, 1.02 | 0.08 |

#### ^†^ Crude model: no adjustment; model 1: adjustment for amount of bright sunshine hours during 2^nd^ trimester of pregnancy

#### Table S4f: Odds ratio (OR) and 95% confidence intervals (95%CI) for gestational diabetes mellitus (GDM) among women prenatally exposed and unexposed to vitamin D fortified margarine adjusted for amount of bright sunshine hours during the second trimester of pregnancy stratified by women’s season of birth

|  |  | **N** | **Model 1^†^** | | |
| --- | --- | --- | --- | --- | --- |
| **Seasons of birth^¥^** | **Exposure** |  | **OR** | **95%CI** | **p-value** |
| Winter |  |  |  |  |  |
|  | Unexposed | 3465 | 1 |  |  |
|  | Exposed | 3214 | 1.02 | 0.72, 1.44 | 0.89 |
| Spring |  |  |  |  |  |
|  | Unexposed | 3722 | 1 |  |  |
|  | Exposed | 3568 | 0.68 | 0.49, 0.93 | 0.02 |
| Summer |  |  |  |  |  |
|  | Unexposed | 3875 | 1 |  |  |
|  | Exposed | 3675 | 0.88 | 0.64, 1.21 | 0.44 |
| Autumn |  |  |  |  |  |
|  | Unexposed | 3793 | 1 |  |  |
|  | Exposed | 3559 | 1.02 | 0.75, 1.38 | 0.91 |

#### ^†^ Model 1: adjustment for amount of bright sunshine hours during whole pregnancy

^¥^Winter: November to January; Spring: February to April; Summer: May to July; Autumn: August to October

#### Table S4g: Odds ratio (OR) and 95% confidence intervals (95%CI) for gestational diabetes mellitus (GDM) among women prenatally exposed and unexposed to vitamin D fortified margarine adjusted for amount of bright sunshine hours during the third trimester of pregnancy

|  |  | **Crude ^†^** | | | **Model 1^†^** | | |
| --- | --- | --- | --- | --- | --- | --- | --- |
| **Exposure** | **N** | **OR** | **95%CI** | **p-value** | **OR** | **95%CI** | **p-value** |
|  |  |  |  |  |  |  |  |
| Unexposed | 14,855 | 1 |  |  | 1 |  |  |
| Exposed | 14,016 | 0.87 | 0.74, 1.02 | 0.08 | 0.87 | 0.74, 1.02 | 0.08 |

#### ^†^ Crude model: no adjustment; model 1: adjustment for amount of bright sunshine hours during 3^rd^ trimester of pregnancy

#### Table S4h: Odds ratio (OR) and 95% confidence intervals (95%CI) for gestational diabetes mellitus (GDM) among women prenatally exposed and unexposed to vitamin D fortified margarine adjusted for amount of bright sunshine hours during the third trimester of pregnancy stratified by women’s season of birth

|  |  | **N** | **Model 1^†^** | | |
| --- | --- | --- | --- | --- | --- |
| **Seasons of birth^¥^** | **Exposure** |  | **OR** | **95%CI** | **p-value** |
| Winter |  |  |  |  |  |
|  | Unexposed | 3465 | 1 |  |  |
|  | Exposed | 3214 | 0.97 | 0.70, 1.34 | 0.84 |
| Spring |  |  |  |  |  |
|  | Unexposed | 3722 | 1 |  |  |
|  | Exposed | 3568 | 0.74 | 0.53, 1.03 | 0.08 |
| Summer |  |  |  |  |  |
|  | Unexposed | 3875 | 1 |  |  |
|  | Exposed | 3675 | 0.83 | 0.61, 1.14 | 0.26 |
| Autumn |  |  |  |  |  |
|  | Unexposed | 3793 | 1 |  |  |
|  | Exposed | 3559 | 1.15 | 0.83, 1.59 | 0.39 |

#### ^†^ Model 1: adjustment for amount of bright sunshine hours during whole pregnancy

^¥^Winter: November to January; Spring: February to April; Summer: May to July; Autumn: August to October

#### Table S5a: Odds ratio (OR) and 95% confidence intervals (95%CI) for gestational diabetes mellitus among women prenatally exposed and unexposed to vitamin D fortified margarine and born during the 15 month wash-out period* (with unexposed as the reference group)

|  |  | **Crude^†^** | | | **Model 1^†^** | | |
| --- | --- | --- | --- | --- | --- | --- | --- |
| **Exposure** | **N** | **OR** | **95%CI** | **p-value** | **OR** | **95%CI** | **p-value** |
|  |  |  |  |  |  |  |  |
| Unexposed | 14,855 | 1 |  |  | 1 |  |  |
| Wash-out | 9,209 | 1.02 | 0.87, 1.21 | 0.78 | 1.02 | 0.86, 1.21 | 0.83 |
| Exposed | 14,016 | 0.87 | 0.74, 1.02 | 0.08 | 0.87 | 0.74, 1.02 | 0.08 |

#### * Born between June 1985 to September 1986

#### ^†^ Crude model: no adjustment; Model 1: adjustment for women’s season of birth

#### Table S5b: Odds ratio (OR) and 95% confidence intervals (95%CI) for gestational diabetes mellitus among women prenatally exposed and unexposed to vitamin D fortified margarine and born during the 15 month wash-out period* (with exposed as the reference group)

|  |  | **Crude^†^** | | | **Model 1^†^** | | |
| --- | --- | --- | --- | --- | --- | --- | --- |
| **Exposure** | **N** | **OR** | **95%CI** | **p-value** | **OR** | **95%CI** | **p-value** |
|  |  |  |  |  |  |  |  |
| Exposed | 14,016 | 1 |  |  | 1 |  |  |
| Wash-out | 9,209 | 1.18 | 0.99, 1.40 | 0.07 | 1.17 | 0.98, 1.40 | 0.08 |
| Unexposed | 14,855 | 1.15 | 0.99, 1.34 | 0.08 | 1.15 | 0.99, 1.34 | 0.08 |

#### * Born between June 1985 to September 1986

#### ^†^ Crude model: no adjustment; Model 1: adjustment for women’s season of birth

#### Table S6: Trends in birth weight between 1983 to 1988

| **Birth year** | **N** | **Mean** | **SD*** | **p-value**† |
| --- | --- | --- | --- | --- |
| 1983 | 4057 | 3444.2 | 550 |  |
| 1984 | 6971 | 3446.1 | 540 |  |
| 1985 | 7240 | 3449.8 | 544 |  |
| 1986 | 7381 | 3443.0 | 543 |  |
| 1987 | 7383 | 3443.4 | 543 |  |
| 1988 | 5048 | 3432.1 | 536 |  |
| Total | 38080 | 3443.6 | 542 | 0.6 |

#### * SD: Standard deviation

†P-value for difference between groups
